# Modeling dual-range atomic interactions with physicochemical principles for molecular force fields

**DOI:** 10.1093/bioinformatics/btag580

**Published:** 2026-08-08

**Authors:** Honghao Wang, Zunlong Liu, Xiangxiang Zeng, Zhaohui Song, Chen Lin

**Affiliations:** Institute of Artificial Intelligence, Xiamen University, Xiamen, Fujian 361102, China; School of Informatics, Xiamen University, Xiamen, Fujian 361102, China; College of Computer Science and Electronic Engineering, Hunan University, Changsha, Hunan 410082, China; Hangzhou Dianzi University, Hangzhou, Zhejiang 310018, China; School of Informatics, Xiamen University, Xiamen, Fujian 361102, China

## Abstract

**Motivation:**

Machine Learning Force Fields (MLFFs) have emerged as promising tools for accelerating molecular dynamics simulations. However, existing approaches often struggle to capture the geometric characteristics of long-range interactions, including distance and direction, remain sensitive to conformational variations, and lack adaptive mechanisms for balancing short- and long-range forces. To address these limitations, we propose GeoNet, a physicochemical-principle-guided framework for modeling dual-range atomic interactions. GeoNet employs geometric attention over atom–fragment bipartite graphs to characterize long-range dependencies, introduces dual-level augmentation to enforce semantic consistency across molecular conformations, and uses an adaptive fusion module to dynamically balance short- and long-range interaction pathways according to local atomic environments.

**Results:**

Extensive experiments show that GeoNet consistently outperforms ten state-of-the-art baselines across the evaluated benchmarks. Moreover, it achieves the smallest model size and the shortest training time, demonstrating both superior predictive performance and computational efficiency.

**Availability:**

The source code is publicly available at https://github.com/XMUDM/GeoNet.

## 1 Introduction

Molecular dynamics (MD) simulations (Car and Parrinello 1985, Wang *et al.* 2024a), which predict atomic motions by calculating interatomic forces and energies over time, play a central role in modeling molecular behavior. Among them, ab initio molecular dynamics (AIMD) (Cellmer *et al.* 2011), where forces are commonly computed from density functional theory (DFT) (Hohenberg and Kohn 1964, Kohn and Sham 1965), offers high accuracy but is severely constrained by its prohibitive computational cost. This limitation in handling large systems and long timescales has motivated the development of machine-learning force fields (MLFFs) (Zhang *et al.* 2018, Behler 2021, Deringer *et al.* 2021, Unke *et al.* 2021, Zhang *et al.* 2022), which aim to deliver near-DFT accuracy with substantially reduced computational overhead.

In molecular systems, atomic interactions are broadly classified as *short-range* (e.g. covalent bonds and exchange repulsion) and *long-range* (e.g. Van der Waals, hydrogen bonds, and electrostatic forces). Early MLFFs, including EGNN (Satorras *et al.* 2021), DimeNet++ (Gasteiger *et al.* 2020), PaiNN (Schütt *et al.* 2021), and NequIP (Batzner *et al.* 2022), focused exclusively on short-range interactions between atoms. Recently, *hybrid approaches* have been proposed to jointly model short-range and long-range interactions. LSR-MP (Li *et al.* 2024) exemplifies such a framework, where long-range interactions are captured by decomposing molecules into functional fragments and enabling message passing between fragments and atoms.

Although hybrid approaches have demonstrated improved accuracy, they still suffer from the following shortcomings.

**Figure 1:**
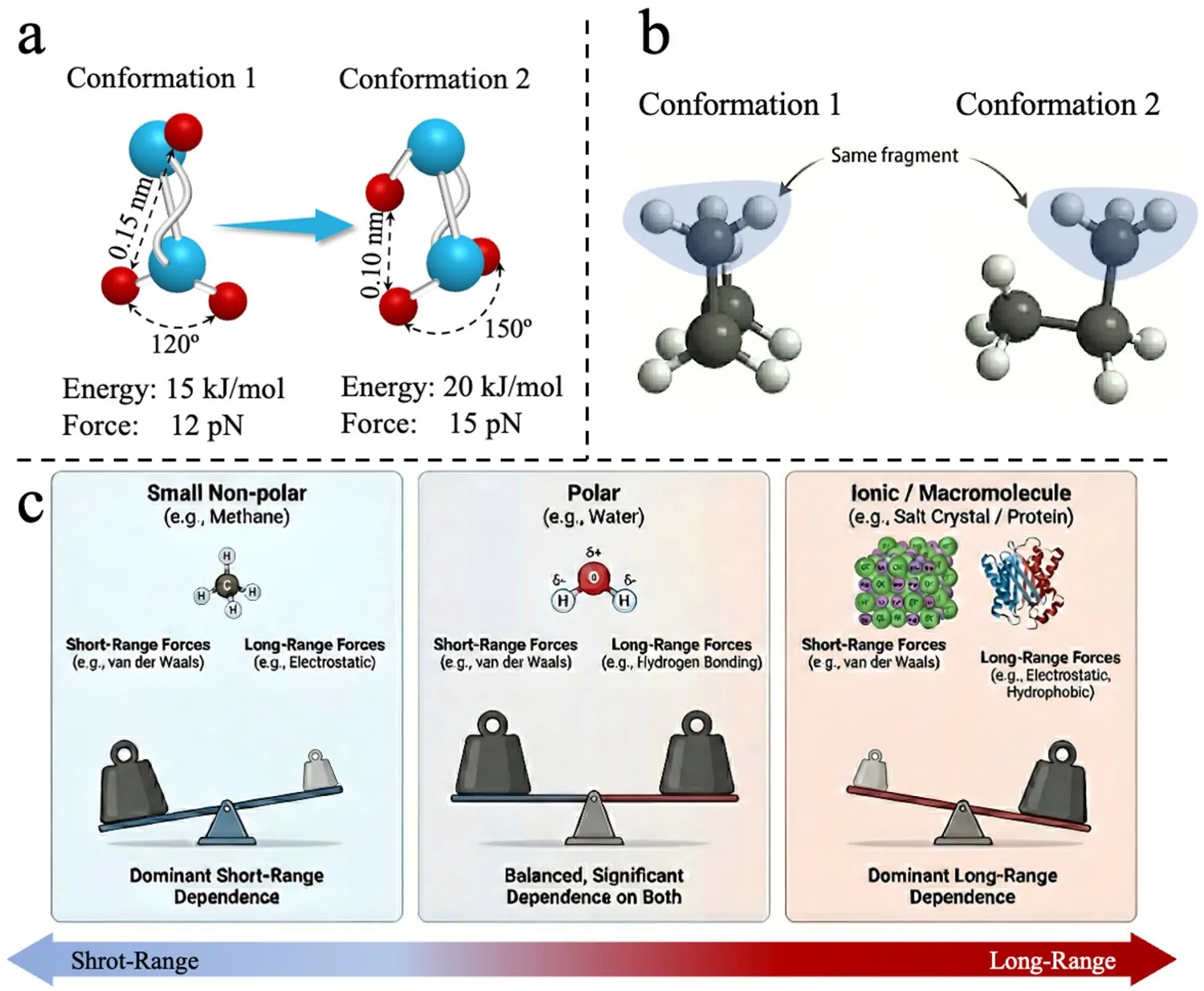
(a) The magnitudes of molecular energies and forces depend strongly on both distances and directions. (b) Identical functional fragments may appear across different molecular conformations. (c) Different molecular systems exhibit varying dependencies on short- and long-range interactions.

1. They fail to capture the physical principles that govern distances and directions. For example, Van der Waals forces, repulsion, and ionic interactions intensify at shorter distances; hydrogen bonds and covalent bonds strengthen when the directional alignment is achieved (Fig. 1a). Thus, these models often perform well only in the vicinity of the training data, but struggle to generalize beyond it.
2. They overlook the impact of molecular conformations. Since different conformations can substantially alter a molecule’s overall dynamical behavior, directly learning from labeled forces and energies tends to yield atomic and fragment representations that vary significantly across conformations. Such variation contradicts the chemical principle that the intrinsic properties of a functional fragment should remain consistent (Fig. 1b), regardless of conformational changes. Consequently, the representation space becomes unstable, leading to distorted and unreliable predictions.
3. Existing models handle short-range and long-range interactions by simply concatenating or summing them, neglecting the fact that different molecular systems exhibit distinct dependencies on these interactions. For example, ionic compounds and polar molecules are more reliant on long-range forces, while non-polar molecules and lipids are more dependent on short-range forces (Fig. 1c). Without a more nuanced integration of these interaction types, models risk misrepresenting the relative contributions of different interactions, leading to inaccurate predictions across molecular systems.

This paper proposes GeoNet, which explicitly models the aforementioned physicochemical properties. Firstly, in modeling the long-range interactions, we propose a novel Geometric Attention mechanism that computes distance-aware and direction-aware attention scores from fragments to atoms, and updates their representations by message passing in the fragment-atom bipartite graph. Secondly, we design a dual-level Conformation-consistent Augmentation strategy to fully exploit the chemical structure. At the atomic level, we perform contrastive learning between atoms before and after molecular-structure augmentation. At the fragment level, we increase similarity among identical fragments across different conformations, while simultaneously encouraging separation between distinct fragments within the same conformation. Finally, we introduce an Adaptive Fusion module that learns to dynamically balance short-range and long-range pathways based on the local physical environment. Collectively, the three innovations naturally embed known rules of molecular dynamics while maintaining low computational cost.

We conduct extensive experiments on two well-known benchmarks comprising seven subsets, and compare our approach against 10 baseline methods (including their variants). GeoNet consistently achieves the best performance in predicting molecular forces and energies.

In summary, our contributions are as follows:

1. We propose GeoNet for MD simulations, a model with strong physical intuition that explicitly incorporates geometric features such as distances and directions to capture long-range interactions, eliminates the influence of different conformations, and learns the varying reliance on long-range and short-range interactions.
2. The proposed model is both accurate and lightweight, reducing the parameter count by approximately *10%* compared with state-of-the-art methods, while delivering an average *12%* performance improvement across multiple benchmark datasets.
3. The proposed model also demonstrates strong robustness. When different short-range interaction modules or fragment separation methods are employed, GeoNet consistently delivers favorable performance.

## 2 Related work

### 2.1 Fragment-based methods

Fragment-based methods decompose molecules into smaller subunits to enable scalable quantum mechanical (QM) computations. Classical strategies include density matrix-based methods (e.g. Divide-and-Conquer (Yang 1991), ADMA (Exner and Mezey 2003), MFCC (He and Zhang 2005)) that reconstruct global electron density from fragments, and energy-based schemes (e.g. MIM (Mayhall and Raghavachari 2011)) that sum fragment energies. In machine learning, methods like LSR-MP (Li *et al.* 2024) integrate rule-based fragments (e.g. BRICS) into graph neural networks for large-molecule representation. GEMS (Unke *et al.* 2024), a fragment-based machine-learned force field framework that combines bottom-up and top-down fragment learning for ab initio–accurate biomolecular dynamics simulations. However, these rely on static fragment definitions and assume semantic consistency across conformations—ignoring geometric variations that cause fragment-level semantic drift.

### 2.2 Long-Range interaction modeling

Standard GNNs struggle with long-range interactions (e.g. electrostatics, dispersion), as their message passing is limited to local neighborhoods. Early solutions added handcrafted potentials or predicted atomic charges, but lacked adaptability. Recent learnable approaches include spatial partitioning, reciprocal-space modeling via Ewald summation (Ewald 1921) or harnessing fast Fourier transformation (e.g. Neural P^3^M (Wang *et al.* 2024b)), and hierarchical graph constructions. Yet most still fuse short-and long-range signals through uniform mechanisms, failing to distinguish their distinct physical natures.

### 2.3 Geometric graph neural networks

Geometric GNNs incorporate 3D structural cues (distances, angles, coordinates) and enforce invariance or equivariance under Euclidean transformations. Invariant models like SchNet (Schütt *et al.* 2017), DimeNet++ (Gasteiger *et al.* 2020), and GemNet (Gasteiger *et al.* 2021) use scalar geometric features for message passing. Equivariant architectures including TFN (Thomas *et al.* 2018), SE(3)-Transformer (Fuchs *et al.* 2020), PaiNN (Schütt *et al.* 2021), and MACE (Batatia *et al.* 2022), explicitly model vector-valued outputs (e.g. forces) with proper rotational behavior. Despite their success in capturing local chemistry, nearly all remain limited to short-range interactions, lacking explicit support for nonlocal effects.

## 3 Method

### 3.1 Overview of GeoNet

Given a molecule with atoms A, its molecular dynamics dataset consists of conformations C. Each c∈C is represented as $<z^{c},P^{c},e^{c},F^{c}>$, where $z^{c}\in R^{\left|A\right|}$ denotes atom IDs, $P^{c}\in R^{|A|\times 3}$ atomic coordinates, and $e^{c}\in R$, $F^{c}\in R^{|A|\times 3}$ the ground-truth energy and forces. GeoNet predicts $\hat{e^{c}}$ and $\hat{F^{c}}$ from $z^{c}$ and $P^{c}$.

**Figure 2:**
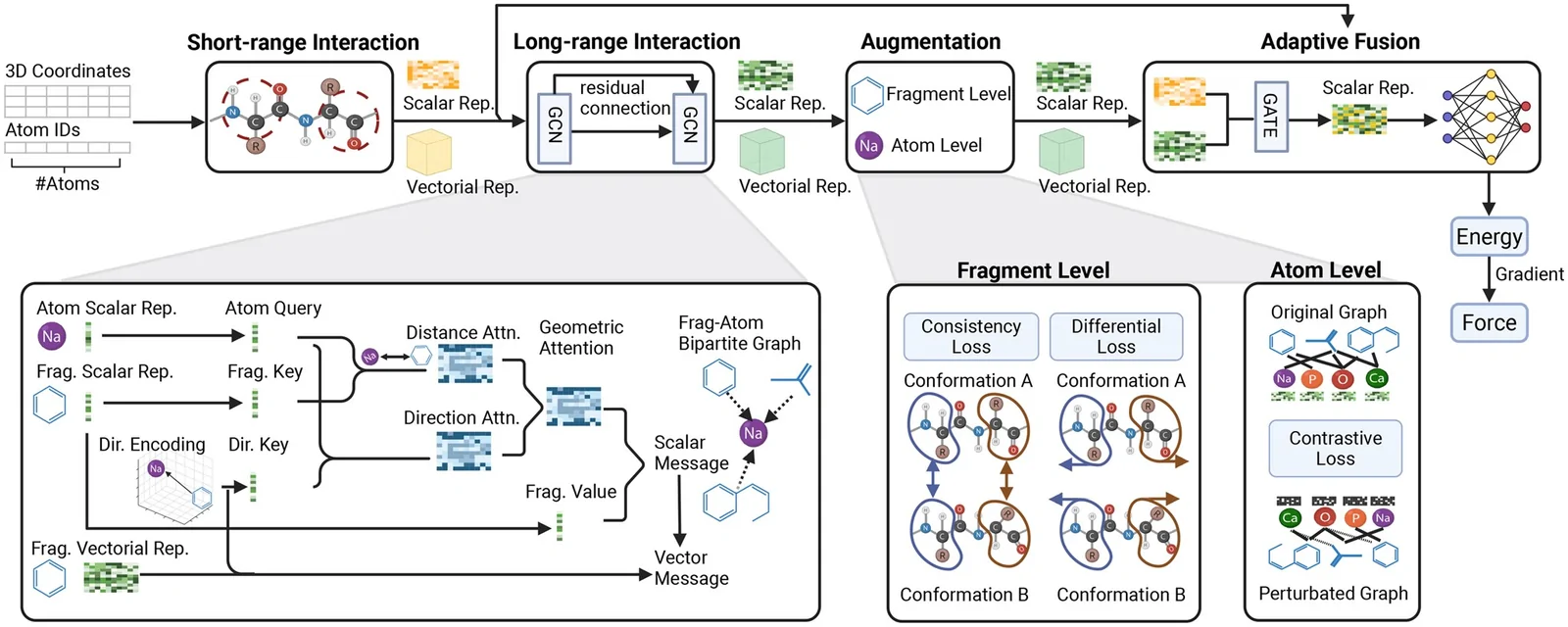
Overview of GeoNet.

Figure 2 illustrates the overall architecture of GeoNet, which jointly models short- and long-range interactions. Atomic identities and coordinates are first encoded by an existing Short-range Interaction backbone to capture local geometric features. The resulting representations are then passed to the Long-range Interaction module, where the Conformation Consistent Augmentation component further enhances long-range interaction modeling. Subsequently, the Adaptive Fusion module adaptively integrates the short- and long-range representations, and the output block predicts the molecular energy and atomic forces.

### 3.2 Long-range interaction

To capture long-range interatomic interactions, this module is based on a Graph Neural Network architecture. Inspired by LSR-MP Li *et al.* (2024), we use existing algorithms to decompose the molecule into chemically meaningful fragments F. By default, we use the BRICS Degen *et al.* (2008) algorithm, but we also evaluate GeoNet ’s performance with respect to different segmentation algorithms in Section 4.

***Graph construction***. First, we construct a bipartite graph $G^{c}=(A,F,E^{c},S^{c})$ to model the interactions between atoms and fragments. The edge matrix $E^{c}\in R^{\left|A|\times |F\right|}$ is defined as $E_{a,f}^{c}=1,\forall a\in A,\forall f\in F$ if $\mathrm{dist}(a,f)\leq r_{\mathrm{long}}$, where $\mathrm{dist}(a,f)=\|P_{a,:}^{c},{\mathrm{mean}}_{a\in f}P_{a,:}^{c}{\|}_{2}$ is the Euclidean distance between the coordinates of atoms and fragments. $r_{\mathrm{long}}$ is the long-range cutoff radius. The distance matrix $S^{c}\in R^{\left|A|\times |F\right|}$ is pre-computed following LSR-MP Li *et al.* (2024) where $S_{a,f}^{c}$ denotes the distance between atom *a* and fragment *f*.

***Initialization of nodal embeddings***. The Short-range Interaction module maps atomic IDs $z^{c}$ and coordinates $P^{c}$ to short-range scalar embeddings $SS^{c}\in R^{|A|\times D}$ and vector embeddings $\vec{VS^{c}}\in R^{|A|\times 3\times D}$, encoding rotation-invariant and rotation-equivariant features, respectively. Based on them, Long-range Interaction maintains long-range scalar representations $SL^{c}\in R^{(|A|+|F|)\times D}$ and vector representations $\vec{VL^{c}}\in R^{(|A|+|F|)\times 3\times D}$ for atoms and fragments, initialized as


(1) $$SL_{a,:}^{c}\leftarrow SS_{a,:}^{c};SL_{f,:}^{c}\leftarrow \mathrm{mean}*a\in fSS_{a,:}^{c}$$

(2) $$V{\vec{L}}_{a,:}^{c}\leftarrow V{\vec{S}}_{a,:}^{c};V{\vec{L}}_{f,:}^{c}\leftarrow \mathrm{mean}*a\in fV{\vec{S}}_{a,:}^{c}.$$

Fragment representations are updated by mean pooling over their constituent atoms. Therefore, we only define f→a message passing to update atomic representations $SL_{a,:}^{c}$ and ${\vec{VL^{c}}}_{a,:}$.

***Geometric Attention***. Since traditional dot-product attention cannot effectively capture internal geometric information, we explicitly model both distance and direction in the attention calculation. As shown in Fig. 2, we define *distance attention* and *direction attention*. Because scalar features are inherently invariant under SO(3) rotations, which ensures that attention weights remain physically consistent with respect to molecular spatial directions, we construct the query $Q_{a,:}^{c}\in R^{D}$ by applying linear projections to the scalar representations of atoms $SL_{a,:}^{c}$.

*Distance attention*. We obtain the fragment key $K_{f,:}^{c}\in R^{D}$ by applying linear projections to the scalar representations of fragment $SL_{f,:}^{c}$, and compute $AD_{a,f}^{c}$ as:


(3) $$AD_{a,f}^{c}=\text{exp}\;\left(-\omega^{\text{dis}}\cdot {\left\|Q_{a,:}^{c}-K_{f,:}^{c}\right\|}_{2}^{2}\right)\cdot S_{a,f}^{c},$$

where $\omega^{\text{dis}}$ is a learnable parameter that regulates the decay rate of the similarity between atoms and fragments.

*Direction attention*. We first encode the direction from *f* to *a* by subtracting their coordinates and normalizing, denoted as $U_{f\to a}^{c}\in R^{3}$.


(4) $$U_{f\to a}^{c}=\frac{P_{a,:}^{c}-{\mathrm{mean}}_{a^{\prime }\in f}P_{a^{\prime },:}^{c}}{{\left\|P_{a,:}^{c}-{\mathrm{mean}}_{a^{\prime }\in f}P_{a^{\prime },:}^{c}\right\|}_{2}^{2}},$$

where ${\mathrm{mean}}_{a^{\prime }\in f}P_{a^{\prime },:}^{c}$ calculates the mean coordinates of the fragment.

Since the long-range vectorial representation ${\vec{VL^{c}}}_{a,:,:}\in R^{3\times D}$ reflects the decomposition of the characteristics of an atom along the three coordinate axes, taking the inner product of ${\vec{VL^{c}}}_{a,:,:}$ and $U_{f\to a}^{c}$ projects the representation of the atom in the direction f→a. We define the *geometric directional feature* $O_{f\to a}^{c}\in R^{D}$ as:


(5) $$O_{f\to a}^{c}=U_{f\to a}^{c}{\vec{VL^{c}}}_{a,:,:}.$$

Then, we compute *direction attention* using the query $Q_{a,:}^{c}$ and the key $K_{f\to a,:}^{c}$ obtained by linearly projecting the geometric feature $O_{f\to a}^{c}$:


(6) $$AR_{a,f}^{c}=\text{exp}\;\left(-\omega^{\text{dir}}\cdot {\left\|Q_{a,:}^{c}-K_{f\to a,:}^{c}\right\|}^{2}\right),$$

where $\omega^{\text{dir}}$ controls the directional sensitivity.

The final *Geometric Attention* $A^{c}$ is formed by fusing the distance and direction components via a learnable gate δ∈(0,1):


(7) $$A_{a,f}^{c}=\mathrm{SiLU}((1-\delta )\cdot AD_{a,f}^{c}+\delta \cdot AR_{a,f}^{c}).$$

***Message passing***. Based on the atom–fragment bipartite graph, we perform message passing using the *Geometric Attention* weights $A_{a,f}^{c}$. Again, since there are two types of representations, we define two types of messages. The *scalar message* from fragment *f* to atom *a* is computed as:


(8) $$M_{f\to a}^{c}=\mathrm{Dense}(A_{a,f}^{c}V_{f,:}^{c}),$$

where $V_{f,:}^{c}\in R^{D}$ is the values obtained by linearly projecting on long-range scalar embeddings $SL_{f,:}^{c}$. Dense(·) is an activated linear layer with bias.

Followed by LSR-MP Li *et al.* (2024), the *vector message* is computed based on the scalar message $M_{f\to a}^{c}$, the directional encoding $U_{f\to a}^{c}$ defined in Eq. 5, and the long-range vector representation ${\vec{VL^{c}}}_{f}$.


(9) $${\vec{M^{c}}}_{f\to a}=M_{f\to a}^{c}⊙\mathrm{MLP}(U_{f\to a}^{c})+M_{f\to a}^{c}⊙\mathrm{MLP}({\vec{VL^{c}}}_{f}),$$

where ⊙ is the Hammard product. MLP(·) is learnable linear functions.

All incoming messages to atom *a* are then aggregated over the neighborhood N(a):


(10) $$M_{a}^{c}=\sum_{f\in N(a)}M_{f\to a}^{c},\;{\vec{M^{c}}}_{f}=\sum_{f\in N(a)}{\vec{M^{c}}}_{f\to a}.$$

Finally, the atomic scalar and vector embeddings are updated via residual connections:


(11) $$\begin{array}{c} SL_{a,:}^{c}\leftarrow SL_{a,:}^{c}+\mathrm{MLP}(M_{a}^{c})⊙{\vec{VL^{c}}}_{a,:,:}+\mathrm{MLP}(M_{a}^{c}),\\ {\vec{VL^{c}}}_{a,:,:}\leftarrow {\vec{VL^{c}}}_{a,:,:}+{\vec{M^{c}}}_{a}+\mathrm{MLP}(M_{a}^{c})⊙{\vec{VL^{c}}}_{a,:,:}. \end{array}$$

This scheme allows the model to propagate both distance- and direction-aware information while maintaining rotation equivariance for the vector representations.

### 3.3 Conformation consistent augmentation

A major drawback of existing studies is their insufficient use of conformational information. Since the dataset contains only a single molecule, fine-grained distinctions are required. Without eliminating the influence of conformations, what is actually learned is a conformational bias, which, in turn, reduces the model’s generalizability. To address this problem, this section proposes novel dual-level augmentation strategies based on inter-fragment and intra-fragment consistency.

***Fragment-level***. Fragments correspond to chemically meaningful functional groups whose intrinsic properties remain relatively stable across conformations. We therefore introduce an *inter-fragment consistency loss* to align embeddings of identical fragments across conformations:


(12) $$L_{\text{inter}}=\frac{1}{|F|\cdot \left(\begin{array}{c} \left|C\right|\\ 2 \end{array}\right)}\sum_{f\in F}\sum_{c,c^{\prime }\in C}(1-\mathrm{sim}(SL_{f,:,:}^{c},SL_{f,:,:}^{c^{\prime }})),$$

where sim(·,·) denotes cosine similarity.

To preserve discriminability among different fragments, we further define an *intra-fragment differential loss*:


(13) $$L_{\text{intra}}=\frac{1}{|F|\cdot \left(\begin{array}{c} \left|C\right|\\ 2 \end{array}\right)}\sum_{f,f^{\prime }\in F}\sum_{c\in C}(1-\mathrm{sim}(SL_{f,:,:}^{c},SL_{f^{\prime },:,:}^{c})).$$

We apply a warm-up schedule to gradually strengthen the consistency constraint:


(14) $$w=w_{\text{init}}+(1-w_{\text{init}})\cdot \text{min}\left(1,\frac{t}{s_{\text{warm}}}\right),$$

where $w_{\text{init}}$ is the initial weight and $s_{\text{warm}}$ the number of warm-up steps. The fragment-level objective is


(15) $$L_{\text{frag}}=w(L_{\text{inter}}+L_{\text{intra}}).$$

***Atom-level***. At the *atom level*, we improve representation robustness by constructing an augmented bipartite graph through feature perturbation and random edge deletion. Let $SS_{a,:}^{c}$ and ${\vec{VS^{c}}}_{a,:,:}$ denote the scalar and vector outputs of the short-range module, and $E^{c}$ the edge set. The augmented representations are defined as:


(16) $$\begin{array}{c} \tilde{SS_{a,:}^{c}}=SS_{a,:}^{c}+\epsilon ,\;,\tilde{{\vec{VS^{c}}}_{a,:,:}}={\vec{VS^{c}}}_{a,:,:}+\lambda ,\\ \tilde{E_{a,f}^{c}}=m_{a,f}^{c}\cdot E_{a,f}^{c},\;m_{a,f}∼\text{Bernoulli}(1-p^{2}), \end{array}$$

where ϵ and λ are zero-mean Gaussian noises with variance $\sigma^{2}$, and $m_{a,f}^{c}$ is the Bernoulli mask for edge dropout with probability *p*. These perturbed inputs are fed into the long-range module (Section 3.2) to obtain $\tilde{SL_{a,:}^{c}}$ and $\tilde{{\vec{VL^{c}}}_{a,:,:}}$.

We form positive pairs between original and augmented representations of the same atom, and negative pairs with augmented representations of other atoms. The contrastive loss is:


(17) $$\begin{array}{c} L_{cl}=-\sum_{N}[\;\sum_{a\in A}\;\text{log}\;\left(\frac{\;\text{exp}\;\left(\mathrm{sim}(SL_{a,:}^{c},\tilde{SL_{a,:}^{c}})/\tau \right)}{\sum_{a^{\prime }\in N}\;\text{exp}\;\left(\mathrm{sim}(SL_{a,:}^{c},\tilde{SL_{a^{\prime },:}^{c}})/\tau \right)}\right)\\ +\sum_{a\in A}\;\text{log}\;\left(\frac{\;\text{exp}\;\left(\mathrm{sim}({\vec{VL^{c}}}_{a,:,:},\tilde{{\vec{VL^{c}}}_{a,:,:}})/\tau \right)}{\sum_{a^{\prime }\in N}\;\text{exp}\;\left(\mathrm{sim}({\vec{VL^{c}}}_{a,:,:},\tilde{{\vec{VL^{c}}}_{a^{\prime },:,:}})/\tau \right)}\right)\;], \end{array}$$

where *N* is the training batch, sim(·) denotes cosine similarity, and τ is the temperature parameter. This objective encourages consistency between original and perturbed atom representations, improving robustness to multi-conformation variations.

### 3.4 Adaptive fusion

Different molecules rely differently on short- and long-range interactions, which simple summation or concatenation cannot capture. We therefore use dedicated subnetworks $\phi_{s}$ and $\phi_{l}$ with a gating network $\psi_{g}$ to adaptively fuse both pathways:


(18) $$\begin{array}{cc} & [\alpha_{s},\alpha_{l}]=\mathrm{softmax}(\psi_{g}(SS^{c}\|SL^{c})),\;[{\vec{\alpha }}_{s},{\vec{\alpha }}_{l}]=\mathrm{softmax}(\psi_{g}(\vec{VS^{c}}\|\vec{VL^{c}})),\\ & U^{c}=\alpha_{s}\cdot \phi_{s}(SS^{c})+\alpha_{l}\cdot \phi_{l}(SL^{c}),\;\vec{U^{c}}={\vec{\alpha }}_{s}\cdot \phi_{s}(\vec{VS^{c}})+{\vec{\alpha }}_{l}\cdot \phi_{l}(\vec{VL^{c}}). \end{array}$$

Here, $\alpha_{s},\alpha_{l}$ and ${\vec{\alpha }}_{s},{\vec{\alpha }}_{l}$ are adaptive fusion weights, and || denotes concatenation. The final scalar representation is obtained by late-stage fusion:


(19) $$h^{c}={\mathrm{DENSE}}_{s}(SS^{c}\|SL^{c}\|U^{c}).$$

The predicted energy is computed by atom-wise aggregation, and forces are derived as its negative coordinate gradients:


(20) $$\hat{e^{c}}=\sum_{a\in A}h_{a}^{c},\;\hat{F^{c}}=-\nabla_{P^{c}}\hat{e^{c}}.$$

Training uses a weighted energy–force MSE objective,


(21) $$L_{t}=(1-\rho )\sum_{c\in C}{\left(e^{c}-{\hat{e}}^{c}\right)}^{2}+\rho \sum_{c\in C}||F^{c}-{\hat{F}}^{c}|{|}_{2}^{2},$$

where ρ balances energy and force supervision. The final loss is


(22) $$L=L_{t}+\mu L_{cl}+\nu L_{\mathrm{frag}},$$

with μ and ν weighting the auxiliary losses.

## 4 Experiment results

In this section, we study the following research questions:

**RQ1:** How does GeoNet compare with state-of-the-art Machine learning force fields (MLFFs) models? (Section 4.2)

**RQ2:** How does GeoNet perform in molecular dynamics simulations? (Section 4.3)

**RQ3:** How does GeoNet perform with different fragment segmentation methods? (Section4.4)

**RQ4:** How does each component in GeoNet perform? (Section 4.5)

**RQ5:** What is the time complexity analysis of GeoNet? (Section 4.6)

### 4.1 Experiments settings

***Datasets***. We evaluated our method on two widely used benchmark datasets, MD22 (Chmiela *et al.* 2023), which contains six different molecules, and Chignolin (Wang *et al.* 2024c). We followed the data partitioning method used in previous work (Chmiela *et al.* 2023, Musaelian *et al.* 2023, Wang *et al.* 2024c), using a 7:1:2 split for training, validation, and testing.

***Evaluation Metric***. We use the mean absolute error (MAE) of the predicted energy and force relative to the DFT reference to assess our method, a standard metric for performance evaluation.

***Hyperparameters***. We use a batch size of 8 and 128 hidden channels. Training adopts a learning rate of $1.00\times 10^{-4}$ with cosine annealing to a minimum of $1.00\times 10^{-7}$. Both the short- and long-range interaction modules contain 2 layers. We apply 1000 warm-up steps and early stopping with a patience of 500. The loss weights are set to $\mu =1.00\times 10^{-4}$, $\nu =1.00\times 10^{-5}$, and ρ=0.5. The short- and long-range cutoffs are 4 Å and 9 Å, respectively. All experiments are conducted on A100 GPUs with training based on standard validation convergence.

### 4.2 Performance comparison

***Competitors***. We compare GeoNet with state-of-the-art baselines, including sgdML (Chmiela *et al.* 2019), PaiNN (Schütt *et al.* 2021), TorchMD-NET (Thölke and De Fabritiis 2022), So3Krates (Frank *et al.* 2022), Allegro (Musaelian *et al.* 2023), MACE (Batatia *et al.* 2022), Equiformer (Liao and Smidt 2023), ViSNet (Wang *et al.* 2024c), Neural P^3^M (Wang *et al.* 2024b) and LSR-MP (Li *et al.* 2024). These baselines are configured with their default hyperparameter settings.

We evaluated two variants of GeoNet incorporating short-range modules adapted from ViSNet and Equiformer, respectively. Specifically, we extracted the **core convolution layers** from these architectures and deployed them with reduced depth (fewer layers). We highlight four observations from Tables 1 and 2:

**Table 1:** Mean absolute errors (MAE) of energy (kcal/mol) and force (kcal/mol/Å) for biomolecules on MD22 and Chignolin.

Dataset	metric	sgdML	PaiNN	TorchMD -NET	SO3Krates	Allegro	MACE	Equiformer	ViSNet	Neural P^3^M	LSR-MP -Equiformer	LSR-MP -ViSNet	GeoNet -ViSNet*	GeoNet -Equiformer*
Ac-Ala3-NHMe	energy	0.3902	0.1168	0.1120	0.3357	0.1014	0.0625	0.0825	0.0791	0.0667	0.0780	0.0654	**0.0593**	0.0714
	force	0.7851	0.2371	0.1867	0.2421	0.1065	0.0874	0.0801	0.0968	0.0912	0.0887	0.0902	**0.0781**	0.0823
AT-AT-CG-CG	energy	1.3872	0.2642	0.2084	0.3458	0.3937	0.1584	0.1520	0.1989	0.1160	0.1335	0.1165	**0.1080**	0.1214
	force	0.7011	0.3702	0.3365	0.3321	0.1284	0.1162	0.1268	0.1567	0.1068	0.1065	0.1083	0.0935	**0.0931**
Buckyball catcher	energy	1.1965	0.3021	0.3824	0.3814	0.5251	0.4815	0.3982	0.4427	0.4327	0.4417	0.4220	**0.2754**	0.4257
	force	0.6816	0.3047	0.2726	0.2312	0.1897	0.1859	0.1117	0.1340	0.1157	0.1130	0.1026	0.0993	**0.0949**
AT-AT	energy	0.7238	0.1675	0.1125	0.1784	0.1431	**0.0759**	0.1317	0.1672	0.0787	0.1007	0.0781	*0.0761* ^#^	0.0952
	force	0.6921	0.2389	0.2039	0.2158	0.0957	0.0998	0.0968	0.1075	**0.0609**	0.0881	0.0795	*0.0747* ^#^	0.0827
DHA	energy	1.3124	0.1158	0.1213	0.3789	0.1157	0.1309	0.1779	0.1531	0.0853	0.0878	0.0873	**0.0812**	0.0824
	force	0.7480	0.1362	0.1209	0.2415	0.0737	0.0648	0.0512	0.0674	0.0609	0.0534	0.0598	0.0523	**0.0481**
Stachyose	energy	4.0507	0.1524	0.1395	0.4423	0.2489	0.1247	0.1401	0.1288	0.1059	0.1252	0.1055	**0.0984**	0.1027
	force	0.6769	0.2321	0.9527	0.4354	0.0975	0.0874	0.0641	0.0873	0.0784	0.0632	0.0767	0.0692	**0.0559**
Chignolin	energy	–	2.4502	2.5304	–	3.5271	–	1.0981	2.4362	2.5931	1.0981	2.4362	2.3157	**1.0243**
	force	–	0.6831	0.6524	–	0.3829	–	0.2132	0.3721	0.3927	0.2072	0.3721	0.3549	**0.1985**
Double–walled nanotube	energy	4.0122	1.1124	1.4732	1.3261	2.2097	1.3725	1.1945	1.0339	0.7759	1.5433	1.8230	0.8872	**0.7507**
	force	0.5231	0.9168	1.0031	0.9941	0.3428	0.8794	0.2747	0.3959	0.2561	0.2972	0.3107	0.3549	**0.2351**

The best one in each category is highlighted in bold black.

* Indicates that we are using the *core convolutional layers* of ViSNet and Equiformer.

# Indicates the second best result.

**Table 2:** Model size and training time.

Methods	ViSNet	LSR-MP -ViSNet	PaiNN	Allegro	Equiformer	GeoNet -ViSNet*
**Parameters**	2.23M	1.8M	3.25M	16.23M	3.11M	**1.6M**
**Training time**	46 s	22s	21s	852s	162s	**17s**

1. Consistent superiority: GeoNet achieves lowest MAE in 12 of 14 tasks and second-best in 2, reducing energy errors by 4.8%–7.1% on molecules like Ac-Ala3, DHA, and Chignolin, and performing well on supramolecules using eMolFrag.
2. Scalability: unlike baselines that struggle on large systems (e.g. >2.5 kcal/mol on Chignolin), GeoNet reduces Buckyball catcher error by 42.8%, capturing global structural information effectively.
3. Architectural universality: stacking GeoNet on backbones like ViSNet or Equiformer consistently improves performance, e.g. reducing Stachyose force errors by 12.8%.
4. Efficiency: GeoNet outperforms LSR-MP variants with fewer parameters and shorter training times, offering a practical, computationally efficient solution.

### 4.3 Efficient and accurate molecular dynamics simulations

**Figure 3:**
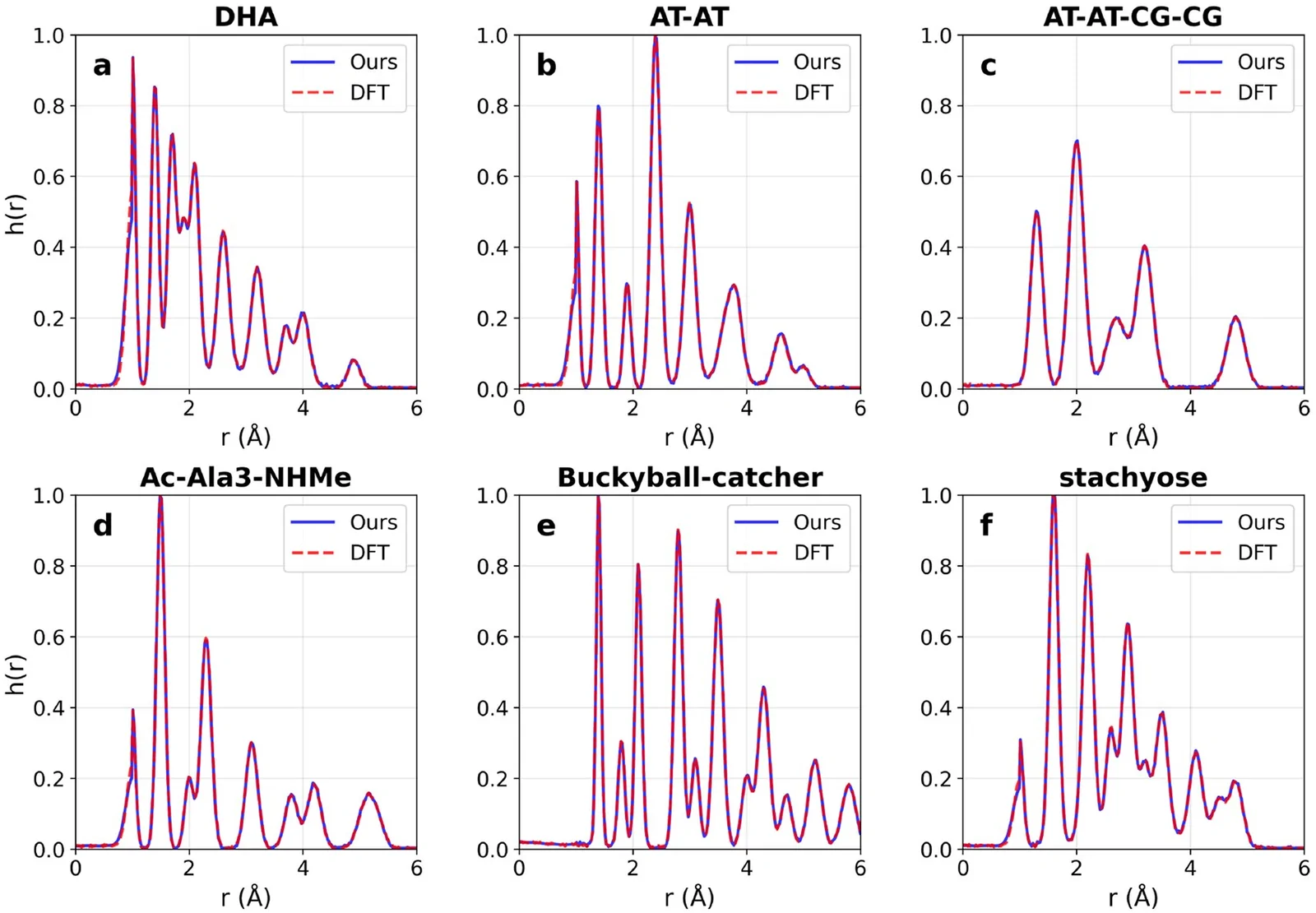
Comparison of interatomic radial distribution functions h(r) computed from molecular dynamics trajectories generated by GeoNet and from ab initio molecular dynamics (AIMD) simulations at the DFT level.

**Figure 4:**
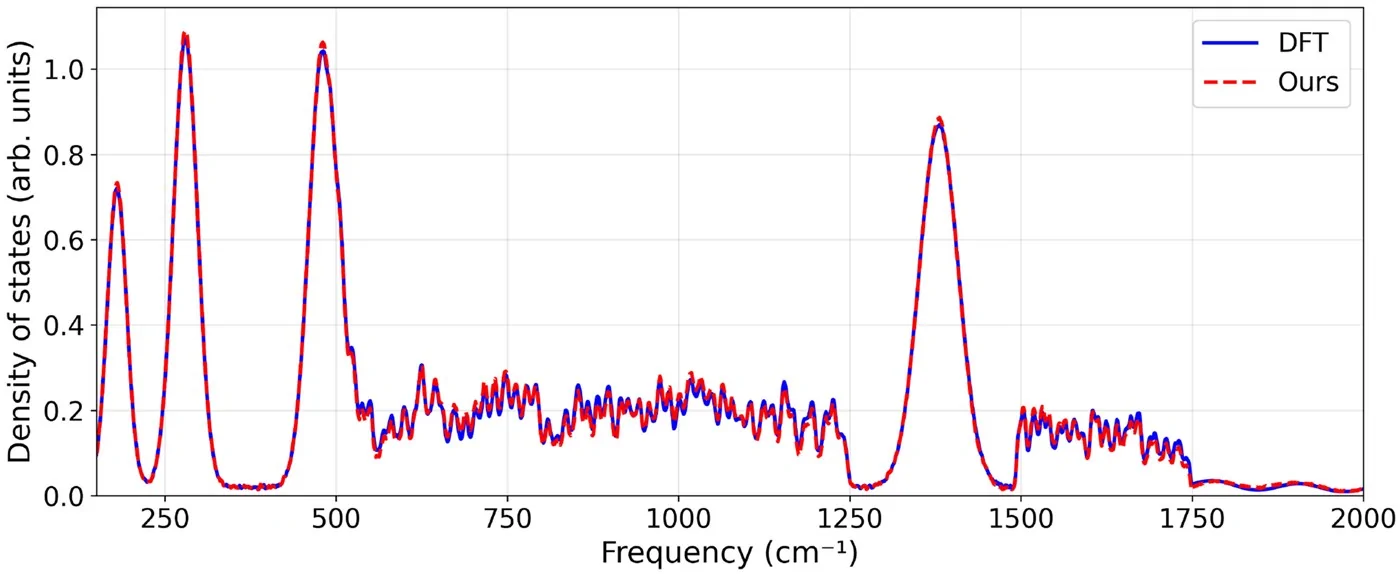
Comparison of spectra from our model and DFT for Ac-Ala3-NHMe.

Table 1 demonstrates GeoNet ’s high accuracy in **energy** and **force** predictions. To evaluate stability in dynamic simulations, we performed molecular dynamics experiments using the MD22 dataset (Chmiela *et al.* 2023) integrated with the Atomic Simulation Environment (ASE) (Larsen *et al.* 2017)]. Short MD simulations with 2000 training configurations, a 0.5 fs time step, and the Berendsen thermostat show that interatomic radial distribution functions h(r) closely match ab initio MD results (Fig. 3), indicating accurate reproduction of atomic arrangements. For long-time behavior, 200-ps NVE simulations on Ac-Ala3-NHMe with a 1 fs time step reveal that GeoNet maintains stable trajectories, preserves energy, and captures major peptide conformations, comparable to DFT potentials (Fig. 4), demonstrating its ability to model long-timescale molecular dynamics.

### 4.4 Robustness under different fragment segmentation algorithms

GeoNet learns long-range representations on an atom–fragment bipartite graph constructed from existing segmentation algorithms. To assess robustness, we compare BRICS(-B), Recap(-R), and eMolFrag(-E). As shown in Table 3, GeoNet consistently outperforms the corresponding LSR-MP baseline under the same fragmentation strategy, reducing energy and force MAE by 5–12% and 4–10%, respectively. This robustness is attributed to Conformation Consistent Augmentation, which aligns fragment-level representations to reduce conformational variance and stabilizes atom-level embeddings via contrastive learning, making the model less sensitive to segmentation choices.

**Table 3:** Performance of GeoNet and LSR-MP under different molecular fragmentation strategies.

Dataset		LSR-MP-B	GeoNet-B	LSR-MP-R	GeoNet-R	LSR-MP-E	GeoNet-E
AT-AT	energy	0.0781	**0.0761**	0.0827	**0.0803**	0.0854	**0.0823**
	force	0.0795	**0.0747**	0.0795	**0.0775**	0.0827	**0.0787**
AT-AT-CG-CG	energy	0.1165	**0.1080**	0.1325	**0.1130**	0.1624	**0.1415**
	force	0.1083	**0.0935**	0.1164	**0.1010**	0.1439	**0.1208**
Ac-Ala3-NHMe	energy	0.0654	**0.0593**	0.0681	**0.0650**	0.0709	**0.0653**
	force	0.0902	**0.0781**	0.0937	**0.0912**	0.1018	**0.0890**
DHA	energy	0.0873	**0.0812**	0.1021	**0.0925**	0.0859	**0.0825**
	force	0.0598	**0.0523**	0.0735	**0.0628**	0.0572	**0.0558**
Stachyose	energy	0.1055	**0.0984**	0.1012	**0.0970**	0.1279	**0.1055**
	force	0.0767	**0.0692**	0.0717	**0.0671**	0.0806	**0.0740**
Chignolin	energy	2.4362	**2.3157**	2.8928	**2.3130**	2.5934	**2.3545**
	force	0.3721	**0.3549**	0.5430	**0.3795**	0.4839	**0.4111**

-B indicates that the model uses the Brics algorithm.

-R indicates that the model uses the Recap algorithm.

-E indicates that the model uses the eMolFrag algorithm.

### 4.5 Ablation study

To investigate the sources of performance gains, we conducted an ablation study based on LSR-MP, incrementally adding the Adaptive Fusion (AF) and Conformation Consistent Augmentation (CCA) modules and replacing conventional attention with Geometric Attention (GA) in the GNNs (Table 4). Results show that Adaptive Fusion significantly boosts performance by decoupling short- and long-range interactions, Conformation Consistent Augmentation improves accuracy by stabilizing predictions across conformations, and Geometric Attention further enhances modeling of complex spatial and distance-dependent interactions. Visualizations confirm that each module effectively fulfills its intended functional role.

**Table 4:** Ablation of performance under different modules.

Dataset		Vanilla	+AF	+AF + CCA	**+AF + CCA** **+GA**
AT-AT	energy	0.0917	0.0823	0.0804	**0.0779**
	force	0.0790	0.0779	0.0768	**0.0747**
AT-AT-CG-CG	energy	0.1653	0.1441	0.1176	**0.1080**
	force	0.1400	0.1281	0.1062	**0.0935**
Ac-Ala3-NHMe	energy	0.1029	0.0907	0.0721	**0.0593**
	force	0.1208	0.1078	0.0935	**0.0781**
DHA	energy	0.1142	0.1013	0.0904	**0.0812**
	force	0.0683	0.0657	0.0613	**0.0523**
Stachyose	energy	0.1458	0.1274	0.1021	**0.0984**
	force	0.0913	0.0802	0.0787	**0.0692**
Chignolin	energy	2.9497	2.7534	2.6471	**2.3157**
	force	0.6913	0.4897	0.4237	**0.3549**

#### 4.5.1 Visualization of geometric attention

**Figure 5:**
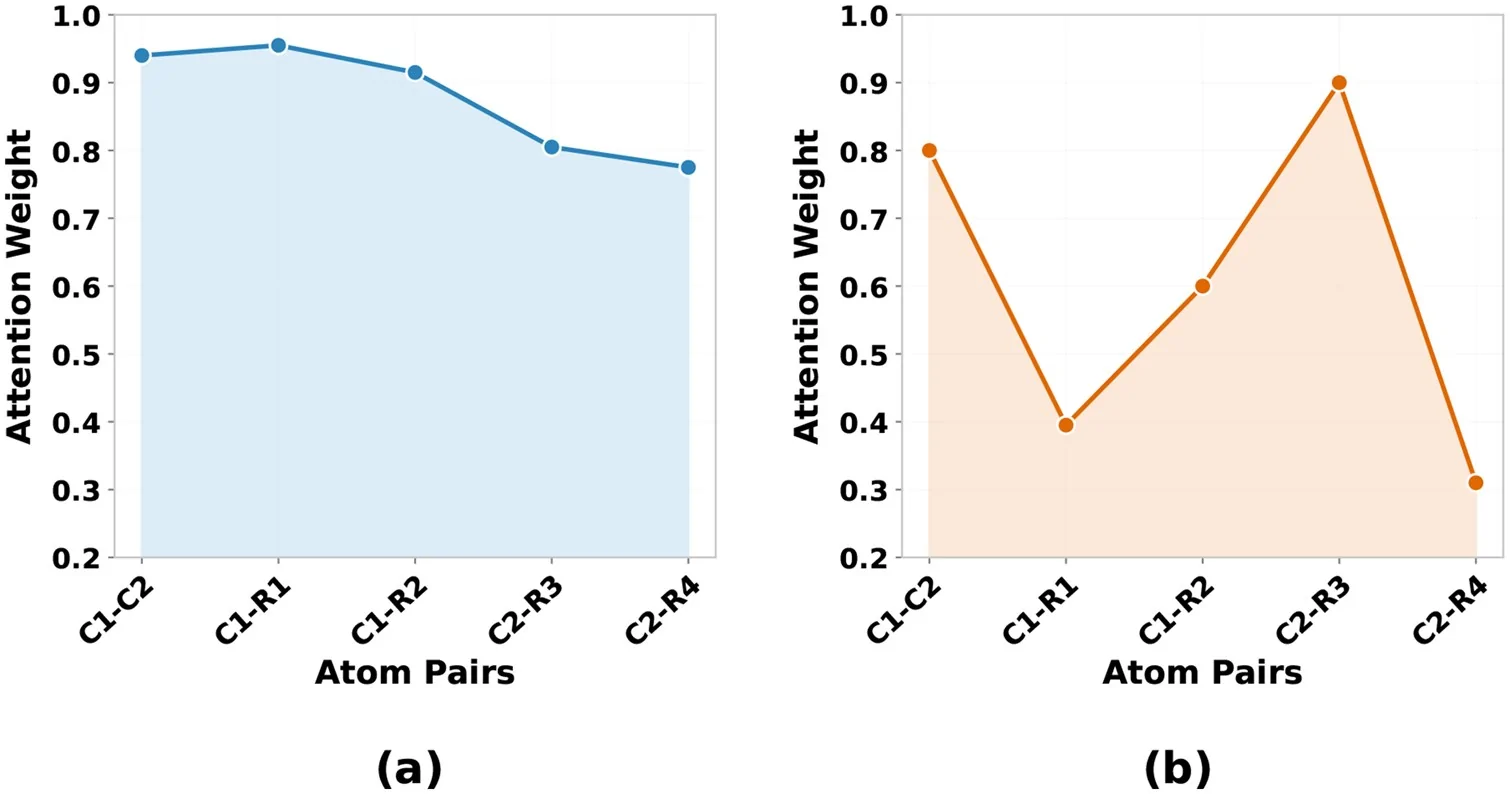
Comparison of attention weight distributions within a molecular conformation. (a) Attention weights produced by conventional attention. (b) Attention weights produced by Geometric Attention.

We visualize attention scores for a random AT-AT-CG-CG conformation in Fig. 5. Traditional attention assigns nearly uniform weights, whereas geometric attention differentiates edges by interatomic distance and orientation, producing distinctive patterns and enhancing spatial sensitivity for more accurate energy predictions.

#### 4.5.2 Visualization of conformation consistent augmentation

**Figure 6:**
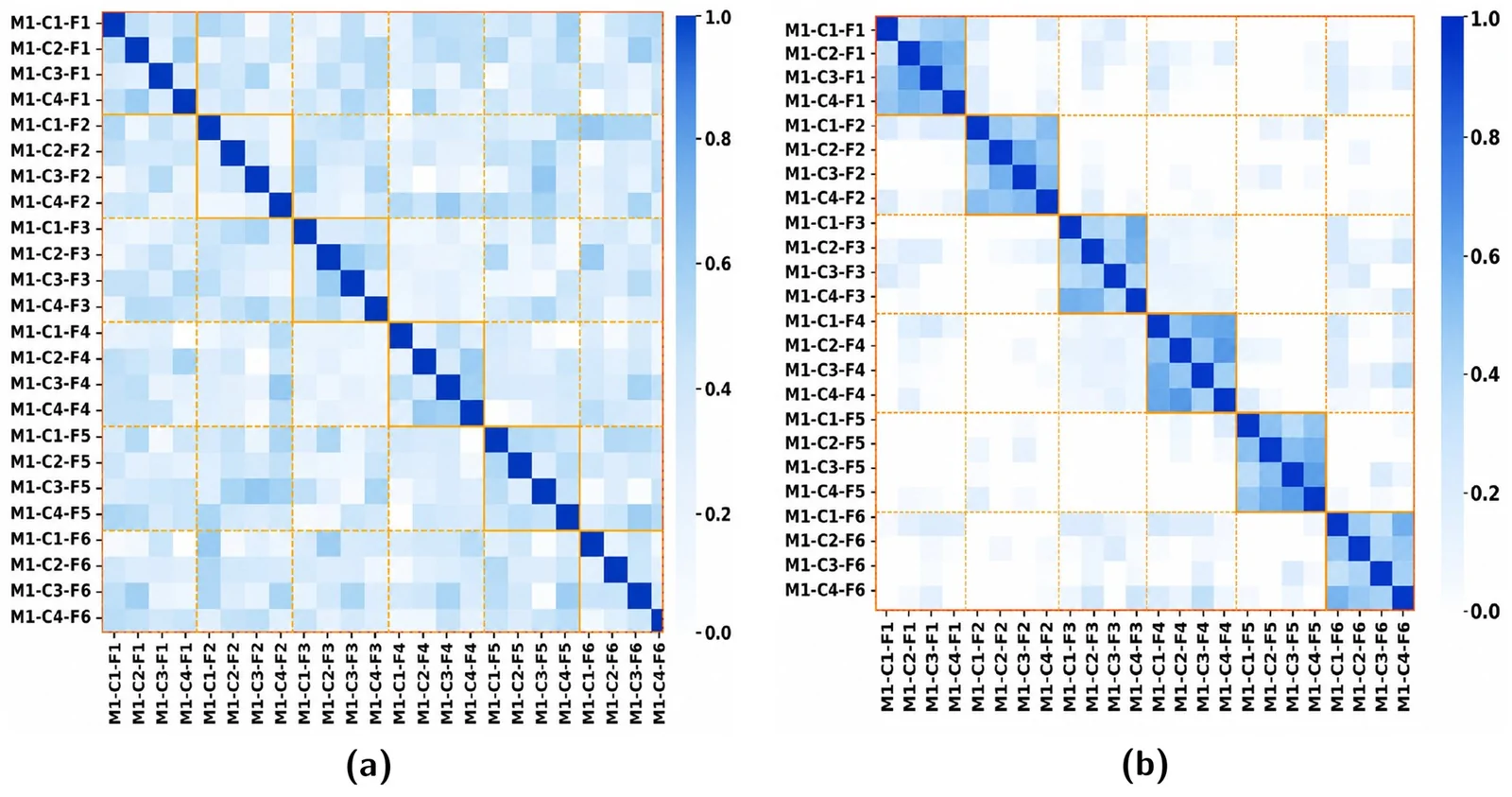
Comparison of fragment representations before and after applying Conformation Consistent Augmentation. (a) Fragment representations obtained without Conformation Consistent Augmentation. (b) Fragment representations obtained with Conformation Consistent Augmentation.

Figure 6 shows fragment embeddings in the M-C-F space (M: molecule, C: conformation, F: fragment). Without Conformation Consistent Augmentation, identical fragments across conformations are widely dispersed; with Conformation Consistent Augmentation, they cluster tightly while remaining distinct from other fragments, demonstrating improved robustness to geometric variations and preserved discriminability.

#### 4.5.3 Ablation of the long-range and short-range cutoff

The cutoff for short- and long-range interactions are initially chosen based on common practices in molecular simulations and is further tuned according to validation performance. We further conduct a sensitivity analysis on the cutoff radius for both short-range and long-range interactions to systematically evaluate its impact on energy and force prediction accuracy.

As shown in Table 5, increasing the long-range cutoff from 6 Å to 9 Å substantially improves performance, while larger cutoffs yield only marginal gains. This indicates that a 9 Å range captures most relevant non-local interactions, whereas further expansion adds limited information, potential redundancy, and higher inference cost, supporting our chosen setting.

**Table 5:** Effect of different long-range cutoffs on performance and inference efficiency on AT-AT-CG-CG.

Long-range cutoff (Å)	Energy MAE (kcal/mol)	Forces MAE (kcal/mol/Å)	Inference Time (ms)
6	0.1217	0.1081	10.11
9	**0.1080**	**0.0935**	**10.24**
12	0.1072	0.0957	10.39
15	0.1097	0.0989	10.41

In Table 6, we observe that the inference time gradually increases as the cutoff radius becomes larger. The MAE decreases when the cut-off radius increases from 2 Å to 4 Å, but rises again when it further increases from 4 Å to 6 Å, indicating that 4 Å provides the best trade-off between accuracy and efficiency.

**Table 6:** Effect of different short-range cutoffs on performance and inference efficiency on AT-AT-CG-CG.

Short-range cutoff (Å)	Energy MAE (kcal/mol)	Forces MAE (kcal/mol/Å)	Inference Time (ms)
2	0.1141	0.1074	10.16
4	**0.1080**	**0.0935**	**10.24**
6	0.1091	0.0943	10.30

### 4.6 Complexity analysis of GeoNet

In GeoNet, the bipartite graph between atoms and fragments introduces O(N×M) connections in the worst case, where *N* is the number of atoms and M≪N is the number of fragments. In practice, both geometric attention and atom–fragment interactions are restricted to local neighborhoods, leading to an effective complexity of O(N×k), where k≪N denotes the number of local neighbors. Thus, the overall computation is more efficient than fully connected atom-level models with $O(N^{2})$ complexity. To evaluate efficiency, we measure wall-clock runtime and report per-step inference time in MD simulations across molecules of increasing size (from Ac-Ala3 to Chignolin).

In Table 7, GeoNet achieves the lowest per-step inference time across all methods and maintains this advantage as system size increases, demonstrating both theoretical efficiency and practical speed in MD simulations.

**Table 7:** Per-step inference runtime (seconds) in MD simulations.

Dataset	VisNet	LSM–MP	NP3M	GeoNet
Ac–Ala3–NHMe	0.0201	0.0152	0.0127	**0.0104**
Chignolin	0.1534	0.0911	0.0743	**0.0621**

Lower is better.

## 5 Conclusion

We introduce GeoNet that explicitly models long-range geometric interactions, mitigates conformational variability to enhance robustness, and adaptively fuses physical effects. Experiments on MD22, Chignolin, and real-world MD simulations demonstrate consistent gains in accuracy, robustness, and efficiency over state-of-the-art baselines. Our work provides new insights into how physicochemical principles can be systematically embedded into MLFFs, suggesting that future research may explore richer geometric constraints.

## Data Availability

The datasets used in this study are publicly available. Details of the datasets and their original sources are provided in Section 4.1 (Experimental Settings) and the corresponding references.
